# Evaluating Accuracy of Smartphone Facial Scanning System with Cone-Beam Computed Tomography Images

**DOI:** 10.3390/bioengineering12080792

**Published:** 2025-07-23

**Authors:** Konstantinos Megkousidis, Elie Amm, Melih Motro

**Affiliations:** Department of Orthodontics and Dentofacial Orthopedics, Henry Goldman School of Dental Medicine, Boston University, Boston, MA 02118, USA

**Keywords:** cone-beam computed tomography, stereophotogrammetry, three-dimensional imaging, facial scanning

## Abstract

Objectives: Facial soft tissue imaging is crucial in orthodontic treatment planning, and the structured light scanning technology found in the latest iPhone models constitutes a promising method. Currently, studies which evaluate the accuracy of smartphone-based three-dimensional (3D) facial scanners are scarce. This study compares smartphone scans with cone-beam computed tomography (CBCT) images. Materials and Methods: Three-dimensional images of 23 screened patients were captured with the camera of an iPhone 13 Pro Max and processed with the Scandy Pro application; CBCT scans were also taken as a standard of care. After establishing unique image pairs of the same patient, linear and angular measurements were compared between the images to assess the scanner’s two-dimensional trueness. Following the co-registration of the virtual models, a heat map was generated, and root mean square (RMS) deviations were calculated for quantitative assessment of 3D trueness. Precision was determined by comparing consecutive 3D facial scans of five participants, while intraobserver reliability was assessed by repeating measurements on five subjects after a two-week interval. Results: This study found no significant difference in soft tissue measurements between smartphone and CBCT images (*p* > 0.05). The mean absolute difference was 1.43 mm for the linear and 3.16° for the angular measurements. The mean RMS value was 1.47 mm. Intraobserver reliability and scanner precision were assessed, and the Intraclass Correlation Coefficients were found to be excellent. Conclusions: Smartphone facial scanners offer an accurate and reliable alternative to stereophotogrammetry systems, though clinicians should exercise caution when examining the lateral sections of those images due to inherent inaccuracies.

## 1. Introduction

The triad of teeth, soft and hard facial tissues play an extensive role in orthodontic diagnosis and treatment planning. Traditional two-dimensional (2D) photogrammetric facial images may exhibit significant inaccuracies attributed to variations in lighting placement, magnification, distortion, and focal length distances. Those limitations of two-dimensional imaging methods have led to increased adoption of three-dimensional (3D) techniques, such as passive and active stereophotogrammetry (SPG), laser-beam scanning (LB), and structured light scanning (SLS).

Among those, SPG systems, which utilize data from multiple images simultaneously taken from different angles extraorally, have found a wide area of application, particularly for soft tissue imaging, which offers advantages such as limited radiation exposure, efficient capturing of facial morphology and soft tissue changes, excellent reproducibility of texture and color, reduced acquisition time, unobstructed 360° views not impacted by the angle and distance between patient and camera, and the ability to be combined with cone-beam computed tomography (CBCT) images [1,2,3,4,5]. Hence, SPG is especially useful in orthodontic and surgical pre- and post-treatment evaluations of dentoskeletal and craniofacial relationships and facial aesthetics considerations [6]. While traditional 3D SPG systems like the stationary “3dMD Face” system (3dMD, Atlanta, GA, USA) are still considered the gold standard for three-dimensional soft tissue evaluations [7], they come with significant drawbacks, including exorbitant costs, system complexity, space needs, and calibration requirements, rendering them impractical for everyday clinical use [8,9,10].

Structured light scanners (SLSs) are an alternative to SPG. An SLS employs active 3D sensors, projecting organized light patterns onto a patient’s extraoral soft tissue. High-resolution cameras capture white, blue, or infrared light patterns, and the distorted light patterns are processed through complex triangulation algorithms, generating a 3D image with additional color texture details [11,12,13].

Incorporating similar SLS technology into portable devices, like smartphones and tablets, presents an opportunity for smartphone-based 3D imaging [14]; one such example is the TrueDepth camera used for facial recognition in the latest iPhones (Apple Inc., Cupertino, CA, USA). This camera has a dot projector that uses structured light and projects 30,000 infrared points on the person’s face in a specific pattern received by the infrared camera. Then, an ambient light projector and optical camera enhance data capture and apply color to each point of the three-dimensional model [15]. This situation allows developers to design applications for 3D scanning by employing open-source scripts and software coding [8,16]. Various companies have created three-dimensional SPG systems, each with unique image-processing software. Hence, it becomes feasible to capture up to 500,000 3D data points and generate a high-definition 3D model of a face within seconds [17,18,19].

As soft tissues are becoming one of the crucial considerations in treatment planning for orthodontists, the shift towards these accessible methods of smartphone-based 3D imaging is beneficial for decreased cost and increased usability and time-efficiency in clinical practice. However, currently, there is a lack of research on the accuracy of smartphone-based 3D facial scanners. In addition, no studies have compared soft tissue measurements derived from these scanners with those derived from CBCT—a standardized method of 3D modeling regarded for its highly accurate 3D surface rendering and reproducibility [20,21,22,23], even compared to extraoral methods [24]. Although CBCT does not capture soft tissue color or texture, it has been widely validated for its geometric accuracy and surface rendering reproducibility. In a controlled setting, CBCT-derived 3D models are considered reliable for evaluating trueness due to their consistency in capturing facial surface contours [20,21,22,23], making them an appropriate benchmark for our comparison study.

Thus, this study aims to assess the accuracy as a combination of trueness and precision (as defined by ISO 5725-1 [23]) of 3D facial scans taken from a smartphone scanner by directly comparing them with CBCT images.

## 2. Materials and Methods

**Figure 1:**
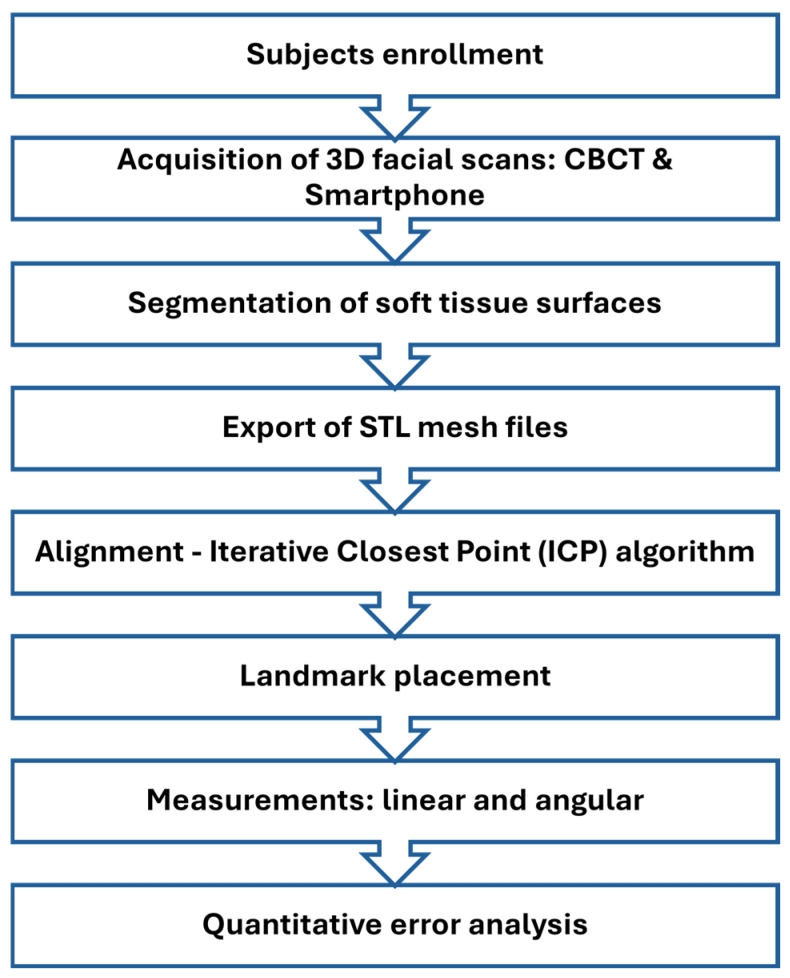
Flowchart summarizing the evaluation process. The study protocol involved subject enrollment, acquisition of 3D facial scans using cone-beam computed tomography (CBCT) and smartphone devices, segmentation of soft tissue surfaces, export of STL mesh files, alignment using the iterative closest Point (ICP) algorithm, landmark placement, linear and angular measurements, and surface deviation analysis to assess accuracy and reproducibility.

The study protocol included participant selection, acquisition of 3D facial scans using both CBCT and smartphone devices, extraction of soft tissue meshes, surface registration, landmark placement, and quantitative error analysis. The following subsections describe each step in detail (Figure 1).

The effect size was derived from the N-Prn-Pg facial angle comparison reported in Akan et al.’s study, which compared measurements between 3dMD and Bellus3D systems [14]. Using G*Power (version 3.1.9.6), we calculated an effect size of 0.17, resulting in a required sample size of 23 subjects (α = 0.05, power = 0.80). Subjects over 18 years of age who require cone-beam computed tomography imaging as a part of their standard of care were included in this study. Subjects with craniofacial syndromes/deformities, trauma history in the maxillofacial area, or facial hair were excluded. Subjects included individuals identifying from three different ethnic groups (nine White participants, six African American, six Hispanic, and two Asian). Participants ranged in age from 18 to 52 years old, with 13 females and 10 males included in the study. Participant ethnicity was self-reported during demographic intake using standardized institutional categories. This information was collected solely for descriptive purposes to reflect the demographic diversity of our sample, with no subgroup analysis performed based on ethnicity.

**Figure 2:**
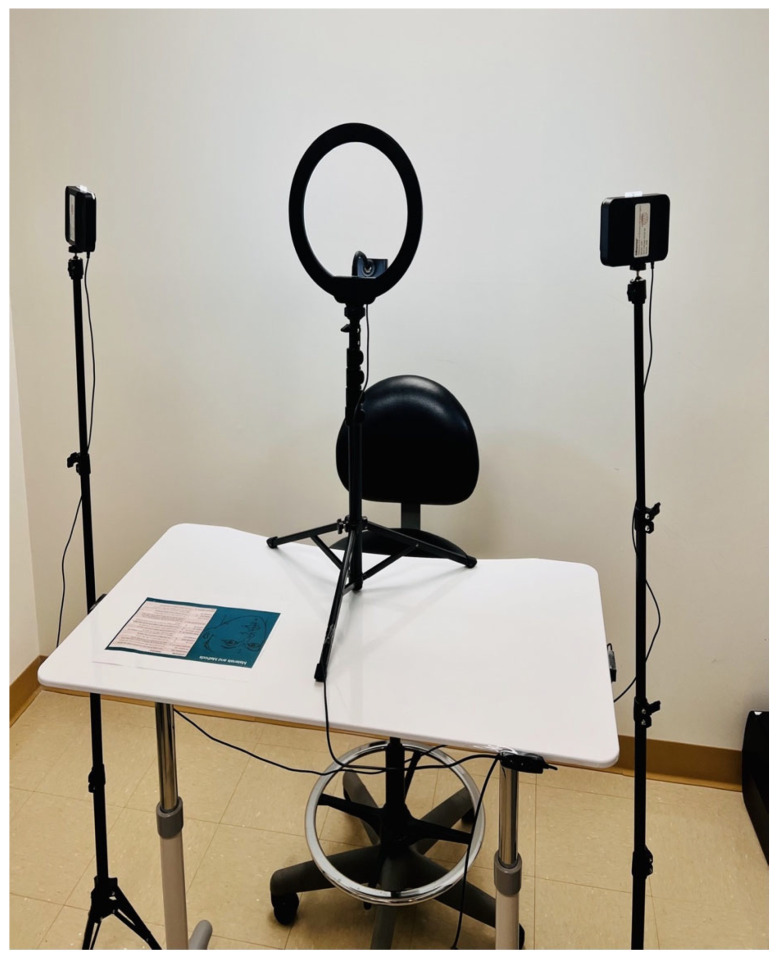
The studio’s configuration is used for smartphone facial scanning.

**Figure 3:**
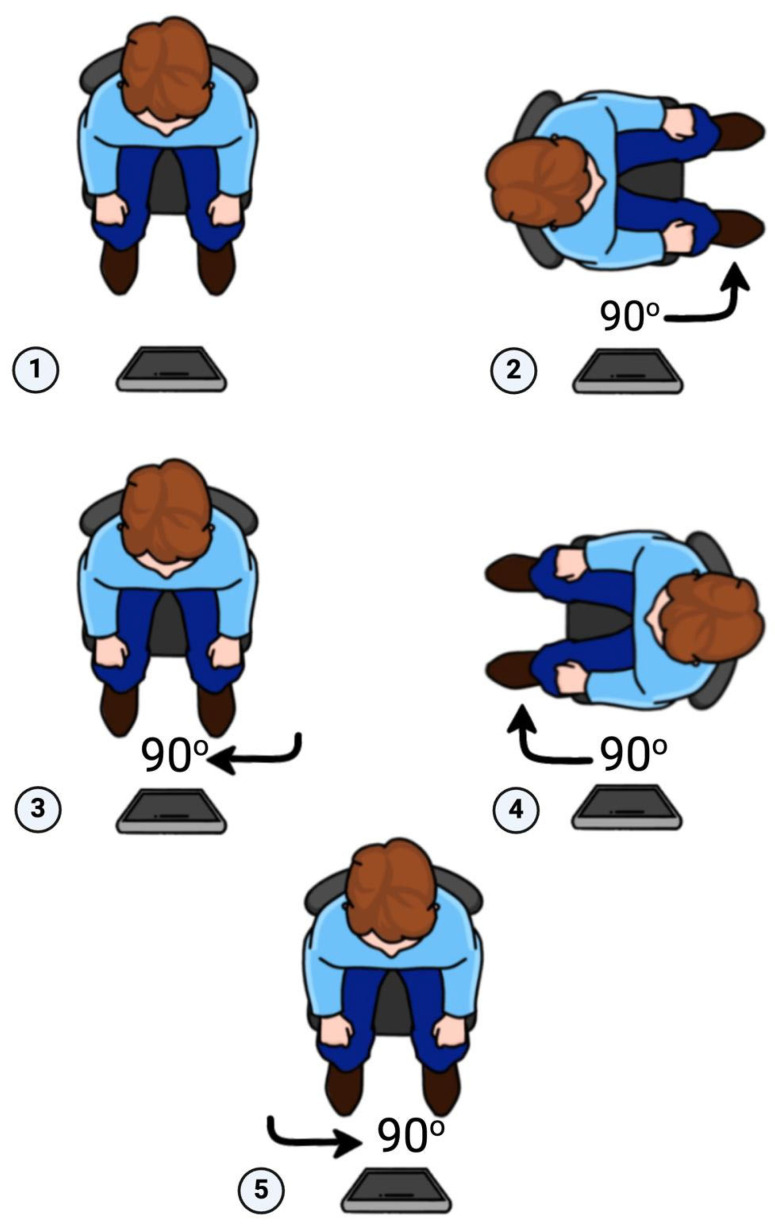
The rotational movements performed during smartphone facial scanning.

During the scanning appointment, 14 soft-tissue anatomic landmarks (Supplementary Table S1), as defined by Farkas [25], were labeled on the subjects’ faces using radiopaque 3 mm dot markers (AliMed, Inc., Dedham, MA, USA) to reduce random errors. Three-dimensional images were captured with the depth of field (TrueDepth) camera of a smartphone (iPhone 13 Pro Max, Apple Inc., Cupertino, CA, USA), and data was processed by a third-party application (Scandy Pro, New Orleans, LA, USA). Digital face scans were taken in the same light settings in a designated room to limit environmental factors. All scans were conducted in a dedicated imaging room under controlled lighting conditions, using a matte, non-reflective background. This setup minimized infrared light reflection and environmental variability, both of which could adversely affect the structured light scanning process and scan fidelity. Patients were asked to swallow, bite down in maximum intercuspation position, and keep their lips in repose position while relaxing their facial muscles and maintaining a neutral expression. Capture was performed by rotating the subjects around the three-dimensional scanner while sitting on a swivel chair against a backrest to maintain an upright head position (natural head position) and prevent unwanted movements (Figure 2 and Figure 3). To maintain a stable head position during smartphone scanning, subjects were seated upright on a swivel chair and gently rotated by an operator standing outside the scanner’s capture field. Care was taken to ensure the operator did not interfere with the projected infrared pattern or introduce background artifacts. The operator’s proximity and positioning were standardized to prevent any influence on scan quality. Each capture lasted approximately 30 s. After each procedure, quality control measures were executed to assess the quality of the scan and check for any facial expression or distortion on the landmarks scanning. Five subjects were randomly selected for a second facial scan (at the same appointment) to assess the device’s reproducibility. Finally, a CBCT scan was acquired using the i-CAT Next Generation system (i-CAT^®^, Imaging Sciences International) as a part of the subjects’ treatment imaging needs. The patients were set up according to the manufacturer’s instructions and asked to maintain the same facial expression and position. The images were obtained after positioning their heads with a head fixation device. A single 360° rotation, 16.5 s scan (tube voltage, 120 kVp; current, 5 mA) with a 17 cm × 23 cm field of view and 0.3 mm isotropic voxel size was then performed on the subjects. Subjects with visible facial hair exceeding 1 mm in length in the perinasal and perioral regions were excluded from this study, as preliminary observations revealed that even short stubble could introduce artifacts in the 3D mesh.

The analysis was based on triangulated surface mesh data exported in STL format from both the smartphone scans and the CBCT reconstructions. These 3D models represent facial geometry with high fidelity and are suitable for registration and landmark measurement. The mesh resolution of the smartphone-derived models was approximately 1 mm, while the CBCT-derived meshes were reconstructed with a voxel size of 0.3 mm using Materialise Mimics. After taking the CBCT and 3D facial scans, postprocessing was performed using the same smartphone application to remove non-anatomical structures from the smartphone scan. This process included cropping the three-dimensional model to remove hair and clothing and the application of a hole-filling function to fill holes in scans smaller than 0.3 mm in diameter. Materialise Mimics software v.24 (version 23.0; Materialise NV, Leuven, Belgium) was used for thresholding and CBCT soft-tissue mask extraction. Then, after establishing unique image pairs of the same patient from the three-dimensional images, they were uploaded to 3-Matic software (version 16.0; Materialise NV, Leuven, Belgium).

Two components of accuracy were evaluated: trueness and precision. Trueness relates to the scanner’s ability to provide 3D reconstruction close to its true form, while precision involves the agreement between images acquired through repeated scanning under the same conditions.

**Figure 4:**
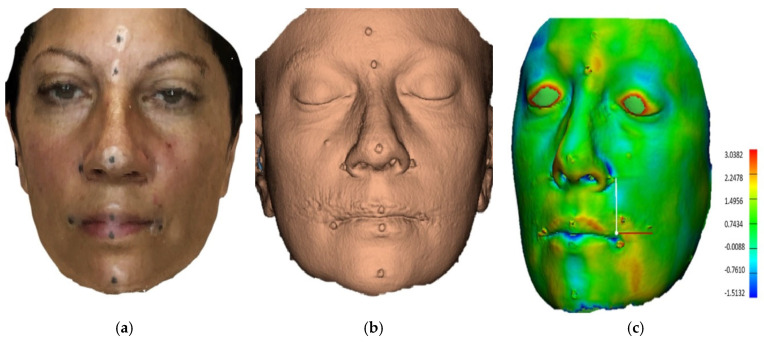
(**a**) Three-dimensional facial image of a sample subject captured using the smartphone scanner; (**b**) corresponding 3D image of the same subject obtained from cone-beam computed tomography (CBCT); (**c**) color-coded surface deviation map illustrating the differences between the CBCT-derived and smartphone-derived 3D facial models after co-registration using the iterative closest point (ICP) algorithm. The color scale represents point-to-point surface deviation in millimeters (mm), with warmer colors indicating greater deviation. Blue areas denote minimal deviation (close to 0 mm), while red areas represent larger discrepancies. All images are aligned in a consistent anatomical orientation to visualize correspondence and assess the trueness of the smartphone scan relative to the CBCT reference.

Following surface extraction, each smartphone-derived 3D mesh was rigidly registered to its corresponding CBCT-derived mesh using the iterative closest point (ICP) algorithm. This algorithm minimizes the root mean square (RMS) of distances between the two surfaces by optimizing translation and rotation. Once alignment was completed, Euclidean distances between corresponding soft tissue landmarks were calculated. These steps were followed to quantify the trueness and reproducibility of the smartphone scanning system. To assess the two-dimensional trueness of the smartphone 3D facial scanner, linear and angular measurements (Supplementary Table S2) in multiple facial areas were compared between the two types of images (Figure 4b,c). Co-registration for each pair was performed using N-point, local, and global registration through the automated iterative closest point method (ICP). For the N-point registration, all midline landmarks were utilized. For the local registration, the forehead, upper nasal dorsum, and zygoma were used for superimposition, as these areas are the most robust regions in aligning different 3D photographs or combining 3D photographs with other imagining modalities such as CBCT [26]. The co-registration of 3D facial models was performed using the iterative closest point (ICP) algorithm in 3-Matic software, with a maximum of 100 iterations and a convergence threshold set at 0.001 mm. These parameters ensured robust and consistent alignment across all image pairs. Following registration, the distance between two 3D virtual models was visualized by generating a heat map (Figure 4c). Soft tissue masks were extracted from CBCT data using thresholding in Materialise Mimics, applying a fixed Hounsfield Unit (HU) range of −200 to +200. This range captured the air-to-soft-tissue interface and was consistently used across all datasets to ensure standardization of segmentation. The quantitative assessment of the scanner’s three-dimensional trueness was determined by the root mean square (RMS) deviation. This procedure was repeated across all 23 pairs of scans. Tolerance thresholds were determined per Aung et al.’s methodology to highlight clinical significance, indicating areas within acceptable clinical error limits [27]. This classification system grouped the point-to-point trueness measures into four categories: (1) highly reliable if the mean difference was <1.0 mm, (2) reliable at 1.0–1.5 mm, (3) moderately reliable at 1.5–2.0 mm, and (4) unreliable if >2.0 mm.

To determine the precision of the smartphone-based facial scanner, a second 3D facial scan was taken on five randomly selected participants right after completing the first capture. The first 3D scan was then compared to the second of the same subject once all linear and angular measurements were also repeated.

Finally, intraobserver reliability was assessed by repeating the same linear and angular measurements described above for five randomly selected subjects after two weeks. All facial scans and subsequent measurements were performed by the same trained operator to ensure methodological consistency. Linear and angular measurements for all participants were completed within a two-week period under identical environmental and equipment conditions.

The normal distribution of the data was evaluated using the Kolmogorov–Smirnov normality test. An independent sample *t*-test was used to determine the significance of the difference between the linear and angular measurements carried out on both three-dimensional images. Descriptive statistics were used to evaluate the superimposition of the images and color map analyses. Intraobserver reliability and scanner precision were assessed by calculating the Intraclass Correlation Coefficient (ICC). This analysis was performed using IBM SPSS Software Version 28.0 (SPSS Inc., Chicago, IL, USA), and statistical significance was assessed using a threshold for type I error of a = 0.05.

## 3. Results

This study found no significant difference in soft tissue measurements between Scandy Pro and CBCT images (*p* > 0.05). The measurement that approached a statistically significant difference was the Prn-Sn-Pg angle (*p* = 0.104). The absolute error, as determined by the mean absolute difference between the Scandy Pro and the CBCT measurements, was 1.43 mm (range = 0.3–3.05 mm) for the linear and 3.16° (range = 0.78–5.08°) for the angular measurement (Table 1 and Table 2).

The comparison of all 3D models obtained by Scandy Pro and CBCT showed that the mean distance and the mean RMS were 0.56 (range = 0.11–1.14) and 1.47 mm (range = 0.96–2.15), respectively. Among the 23 participants, only 2 showed highly reliable results (RMS < 1 mm), 11 had reliable (RMS, 1.0–1.5 mm) results, and 8 had moderately reliable results (RMS, 1.6–2.0 mm). In contrast, two of the participants were found to demonstrate an unreliable registration result (RMS > 2 mm) (Table 3). The mean ICCs of the measurements obtained via Scandy Pro and CBCT were 0.994 and 0.989, respectively, indicating high intrarater reliability (range = 0.97–1). Intrascanner comparison for all assessments showed a high correlation for the Scandy Pro system (ICC = 0.95; range = 0.78–1).

## 4. Discussion

Currently, no other study utilizes CBCT images as a measure of trueness, as most research focuses on direct anthropometry or 3D stereophotogrammetric systems, such as the 3dMD system [28,29,30]. Studies have indicated that CBCT-derived facial scans can be affected by soft tissue movement during scanning, potentially compromising accuracy. This is particularly evident when scanning living subjects, as involuntary movements can introduce artifacts. For instance, research has shown that while CBCT provides detailed anatomical structures, its accuracy in capturing soft tissue can be limited due to motion artifacts [31]. Even though CBCT-derived facial scans may lack true color and surface texture, they are proven to provide high 3D surface accuracy and reproducibility [20,21,22], and were thus optimal in assisting in the determination of trueness in this study. Some studies asserting the accuracy and reproducibility of CBCT-derived facial scans have utilized mannequins to eliminate variables like movement. This approach ensures a controlled environment, free from the challenges posed by patient movement, thereby providing a benchmark for CBCT’s potential accuracy under ideal conditions [32]. It is also important to note that while most studies evaluating 3D facial scanning through smartphone structured light technology utilized the Bellus3D, showing positive outcomes, the manufacturer opted to discontinue the Bellus3D app entirely by the conclusion of 2022 [17,28,29,30,31,32,33]. As a result, the app is no longer accessible for download from the App Store, and as such, we opted for Scandy Pro to be used in our study as one of the most commercial alternatives.

Utilizing the classification thresholds defined by Aung et al. [27], clinicians can evaluate the clinical applicability of 3D scanners based on their RMS deviation, which is particularly critical in applications requiring submillimetric accuracy, such as orthognathic surgery. Even though our study found no statistically significant differences in soft tissue measurements between Scandy Pro and CBCT images (*p* > 0.05), the absolute error determined by the mean absolute difference between the Scandy Pro and the CBCT measurements was 1.43 mm. Among these interlandmark distances, all the measurements at the middle face demonstrated good trueness (<2 mm). However, linear measurements involving lateral landmarks such as Ex(R)-Ex(L), Tr(L)-Tr(R), and Go(L)-Go(R) revealed mean differences of more than 2 mm (2.12, 3.05, and 2.53 mm, accordingly).

These findings agree with those of Andrews et al., who showed significant deviation in the face for structures furthest away from the midline, such as gonion and tragion, which revealed errors outside the 2 mm limit [29]. The authors attributed that to the stretching of soft tissues caused by the movement of the head during the image capture; however, in our study, the subjects remained still; they were rotated on a chair instead. Hence, the discrepancies in the lateral portions of the scans can be attributed to the lens the camera uses. Pinhole cameras lack lenses, resulting in rectilinear images where light travels through the aperture in straight lines; this produces a geometrically precise, replicated inverted object on the image plane. However, in real-life applications, lenses are added to let more light in, which causes radial distortions. The amount of radial distortion changes with increasing the distance from the center of the image, which manifests in the captured images since they undergo unconventional bending to reach the sensor. Then, to make those warped images rectilinear, a calibrated set of coefficients is used, and those characterize the lens’ distortions. Moreover, depth values estimated by the camera at objects’ contours are unreliable (i.e., when the angle between the normal of a point and the viewing ray grows). Lateral noise grows proportionally with distance, whereas axial noise follows a quadratic increase. Both lateral and axial noise show relatively little dependence on the surface angle until around 70 degrees, beyond which they experience a rapid surge [34]. Regarding the lateral canthal measurement, there could be two explanations for the clinically unacceptable results: (1) changes in facial expressions and rapid eye movements (many subjects closed their eyes during CBCT), as has been described previously in the literature and confirmed by Rudy et al. [35], as well as Akan et al.’s results [14], and (2) the inferior ability of the smartphone scanner to capture areas of complex anatomy, such as the eyes and lips [30].

When analyzing the angular measurements, Prn-Sn-Pg and Prn-Sn-Ls were not captured accurately, within a deviation of 1 degree (5.08 and 3.62, respectively), which, according to previous research, has been defined to be the limit for highly reliable measurements [7]. Unlike N-Prn-Pg (which demonstrated a clinically insignificant deviation of 0.78 degrees), the other two angles had in common the subnasal landmark, where the structured light is hard to reach, as also confirmed by Liu et al. [28]. Moving the scanner around the subject, which would allow for easier access to challenging regions to capture, was impossible since the smartphone was fixed on a tripod. This was also observed by Heike et al., who suggested leaning the head back slightly to capture the subnasal and submental regions [36].

Kühlman et al. investigated the accuracy of Scandy Pro on an iPad Pro 2020 against manual anthropometric measurements and found a mean trueness value of 0.47 ± 0.44 mm [37]. One potential reason for these observations and the discrepancy from this study’s results could be that the researchers utilized a mannequin’s face. This choice assists in mitigating errors associated with both involuntary and voluntary movements of subjects. Nevertheless, it can lead to overestimation of the trueness and precision of scanners in clinical settings. This is because patients cannot maintain the same level of absolute stillness as mannequins, especially when the average scan time is approximately 30 s [38]. Moreover, Kühlman et al. only measured linear distances without any 3D analysis [37]. Even though linear and angular measurements are still the most common methods for diagnosis and treatment planning [22], such measurements cannot reflect the 3D geometry of each scan.

This study noted that the mean RMS error for all participants was 1.47 ± 0.34 mm, different from the CBCT facial images. In a recent study by Loy et al., Scandy Pro used on an iPhone 12 Pro had reliable 3D trueness with a 3D Head RMS Error of 1.41 [39], which agrees with our results. However, it is also crucial to mention that two of our participants were found to demonstrate clinically unacceptable trueness (RMS > 2 mm). Rudy et al. compared 16 pairs of 3D virtual models and found that the iPhone X using Scandy Pro had an RMS value of 0.44 ± 0.10 mm following color map analysis, indicating results superior to the ones acquired in this study [35]. A possible explanation for that is the overlooked fact that Apple has significantly degraded the TrueDepth camera hardware (smaller in size) of the iPhone 13 lineup compared to all previous iPhones, leading to a degradation of depth quality, a sparser dot pattern, and less geometric detail, as confirmed by app developers [40]. Indeed, the images produced by Scandy Pro exhibited notably lower resolution compared to those generated by CBCT. Smartphone scans appeared less realistic and displayed a somewhat rough texture. Despite this visual difference, it is essential to note that the diminished appearance did not impact the accuracy of the 3D shape of the scanned surfaces.

Regarding the precision of the smartphone-based facial scanner, a second 3D facial scan was taken on five randomly selected participants right after completing the first capture, and the ICC values revealed a high correlation (0.95) for the Scandy Pro system, proving good scanning reproducibility, as confirmed by other authors as well [36,40,41,42].

In this study, we employed adhesive radiopaque landmarks to minimize identification errors while performing the measurements. Despite their relatively big diameter (0.3 mm), a circle of equal diameter was used while employing those landmarks for our measurements through the software, thus ensuring accurate identification of these markers’ centers. Indeed, the intraexaminer reliability was rated as excellent (0.99), suggesting that this study’s type of facial markers provides reproducible measurement among different examiners [32].

Concerning the practical implications of this study’s findings, it is recommended that orthodontists integrate this smartphone scanner into their standard set of records. Its affordability, portability, and accessibility make it an attractive option for clinicians. Training requirements are also minimal without any need for additional equipment or space, contributing to workflow efficiency. While it may not be suitable for scenarios requiring precision within 1 mm, such as in orthognathic surgery cases, it holds significant value for assessing dentoskeletal and craniofacial relationships before and after orthodontic treatments, as well as for evaluating growth modification interventions and normal changes over time. For comprehensive treatment planning, especially in orthodontics and craniofacial surgery, integrating facial scans with CBCT and intraoral scans is crucial. This integration allows for a holistic view of both hard and soft tissues, facilitating accurate diagnostics and treatment strategies. Recent advancements have demonstrated methods to effectively merge these datasets, enhancing clinical workflows [43]. Additionally, it presents a promising avenue for telemedicine applications, facilitating improved communication with patients and enabling virtual assessment of treatment outcomes (e.g., clear aligner treatment).

One of the limitations of this study is the scanning time (around 30 s), which allows for minor movements and changes in the participants’ facial expressions, leading to artifacts and inaccuracies. The accuracy of facial scans obtained via smartphones can be compromised by patient movement during the scanning process. Maintaining a static facial expression for the required duration is challenging, leading to potential artifacts and inaccuracies in the captured data. This limitation is acknowledged in studies exploring the feasibility of smartphone-based facial scanning for dental applications [33]. This is especially important in patients with neurological disorders (e.g., Parkinson’s disease) or when patients have difficulty remaining still (e.g., children). Future advancements in real-time video-based scanning may provide a solution for capturing dynamic states with greater precision. Also, our participants were required to move to a completely different area to take the CBCT after the facial scan, further increasing the chances of involuntary facial muscle movement. However, the operator’s experience using the smartphone scanner, providing participants with specific instructions regarding their head position and facial expressions, and using a swivel chair to imitate a purely rotational movement helped minimize this error. Moreover, age, ethnicity, skin tone, gender, and Body Mass Index (BMI) were not considered and could have influenced our measurements; however, that was out of the scope of this study. Finally, our researcher was not blinded to the type of facial images (smartphone vs. CBCT) during the measurements, and interobserver reliability was also not assessed.

To increase clinical utility, seamless integration of smartphone-based facial scans with CBCT and intraoral scans is essential. Current imaging systems and software advancements provide potential pathways for such integration, enabling comprehensive facial and dental analysis. Future studies should prioritize this aspect to facilitate clinical adoption. In addition, future studies should explore how the accuracy of this smartphone scanner may vary depending on the type of mobile phone and application, as well as the scanning protocol utilized. Also, the effect of patient demographics on the scanner’s accuracy will need to be investigated. Finally, researchers should consider evaluating the potential of a longitudinal approach that could offer valuable information regarding the utility of facial scanners in clinical settings and their ability to track changes in soft tissue over time.

Future investigations should explore the integration of smartphone-based scanners with artificial intelligence for automatic landmark recognition and enhanced soft tissue segmentation. The development of dynamic capture systems may also allow for more accurate scanning in patients unable to maintain a static facial posture. Furthermore, seamless integration of facial scans with CBCT and intraoral data within digital treatment planning platforms will enable more comprehensive diagnostics and improve interdisciplinary communication. These advancements may also facilitate remote patient monitoring and virtual treatment simulations in teleorthodontics.

## 5. Conclusions

The results of this study suggest the following:

1. This smartphone scanner’s trueness is clinically acceptable for diagnosis and treatment planning, as the errors were of less than 2 mm in 91% of the subjects.
2. The precision of this system was excellent, demonstrating scanning repeatability.
3. The intraobserver agreement was rated as excellent, indicating the reliability of measurements conducted by the operator.
4. This smartphone facial scanner offers three-dimensional scanning, which is a clinically acceptable alternative to traditional three-dimensional imaging systems. However, clinicians should be cautious when considering the lateral portions of those 3D images due to inherent inaccuracies.

## Figures and Tables

**Table 1:** Linear interlandmark measurements (mean ± Standard Deviation (SD)) comparing cone-beam computed tomography (CBCT) and smartphone 3D scans. *p*-values derived from paired *t*-tests.

Linear Measurements	ScandyPro		CBCT		Mean Absolute Diff	95% CI of the Diff: Lower	95% CI of the Diff: Upper	*p*
	Mean	SD	Mean	SD				
N-Pg (mm)	107.91	7.03	109.2	7.44	1.29	−6.05	2.54	0.416
N-Sn (mm)	55.47	5.80	56.65	5.79	1.18	−4.91	1.97	0.435
Sn-Pg (mm)	53.78	5.93	54.83	5.58	1.05	−4.48	2.37	0.538
Sn-Sl (mm)	39.37	5.11	40.53	5.43	1.16	−4.29	1.97	0.461
Ls-Li (mm)	14.21	3.87	14.51	3.94	0.3	−2.62	2.01	0.793
Ch(R)-Ch(L) (mm)	58.84	7.5	59.68	7.1	0.84	−5.17	3.5	0.701
Al(R)-Al(L) (mm)	44.17	5.16	45.61	5.45	1.44	−4.59	1.72	0.365
Ex(R)-Ex(L) (mm)	94.71	7.4	96.83	7.16	2.12	−6.95	1.7	0.229
En(R)-En(L) (mm)	35.92	3.49	36.67	3.63	0.75	−2.87	1.36	0.477
Tr(L)-Tr(R) (mm)	145.78	9.79	149.38	9.33	3.05	−9.29	2.08	0.358
Go(L)-Go(R) (mm)	126.5	13.11	129.03	13.54	2.53	−10.44	5.39	0.524

**Table 2:** Angular measurements (mean ± Standard Deviation (SD)) of craniofacial reference angles from cone-beam computed tomography (CBCT) and smartphone scans.

Angular Measurements	ScandyPro		CBCT		Mean Absolute Diff	95% CI of the Diff: Lower	95% CI of the Diff: Upper	*p*
	Mean	SD	Mean	SD				
Prn-Sn-Ls (°)	127.15	12.82	130.77	9.56	3.62	−10.33	3.10	0.284
N-Prn-Pg (°)	134.25	8.46	133.47	8.45	0.78	−4.24	5.80	0.756
Prn-Sn-Pg (°)	137.5	11.04	142.58	9.62	5.08	−11.23	1.08	0.104

**Table 3:** Root mean square (RMS) and surface deviation values obtained from color map registration between cone-beam computed tomography (CBCT) and smartphone scans for each participant.

Subject	Average Distance	RMS	Maximum Distance	
			Negative	Positive
1	0.65	1.54	−0.78	5.14
2	0.67	1.4	−1.39	4.57
3	0.32	1.12	−1.87	3.26
4	0.49	1.93	−5.07	4.84
5	0.91	1.84	−2.09	5.34
6	1.14	2.15	−3.7	6.12
7	0.17	1.11	−3.22	3.54
8	1.11	1.91	−1.47	5.37
9	0.4	1.69	−2.71	4.88
10	0.61	1.51	−2.99	3.94
11	0.94	2.05	−4.02	5.91
12	0.43	1.36	−4.47	4.09
13	0.48	1.27	−1.77	3.81
14	0.55	1.13	−1.51	3.03
15	0.84	1.77	−2.64	5.86
16	0.48	1.38	−4.32	3.34
17	0.4	1.35	−1.79	4.43
18	0.54	0.98	−0.89	3.05
19	0.56	1.21	−2	4.12
20	0.11	1.76	−5.18	5.12
21	0.18	1.44	−3.71	3.89
22	0.35	0.96	−1.15	2.78
23	0.56	1.15	−2.67	3.25

## Data Availability

The original contributions presented in this study are included in the article. Further inquiries can be directed to the corresponding author.

## Supplementary Materials

The following supporting information can be downloaded at: https://www.mdpi.com/article/10.3390/bioengineering12080792/s1, Table S1. Soft-tissue anatomic landmarks. Table S2. Linear and angular measurements.
