# Supplementary material for: Evaluating Accuracy of Smartphone Facial Scanning System with Cone-Beam Computed Tomography Images
Source: Bioengineering (Basel). 2025 Jul 23;12(8):792. doi: 10.3390/bioengineering12080792 (PMC12383923; doi:10.3390/bioengineering12080792)
Supplement: Supplementary file 1 [file bioengineering-12-00792-s001.zip › bioengineering-3748314-supplementary.pdf]

bioengineering

MDPI

Supplementary Materials

Evaluating Accuracy of Smartphone Facial Scanning System with Cone-Beam Computed Tomography Images

Konstantinos Megkousidis, Elie Amm * and Melih Motro

Department of Orthodontics and Dentofacial Orthopedics, Henry Goldman School of Dental Medicine, Boston University, Boston, MA 02118, USA; megk@bu.edu (K.M.); mmotro@bu.edu (M.M.)
* Correspondence: elieamm@bu.edu

Table S1. Soft-tissue anatomic landmarks.

**endocanthion (en)**	**Point located at the inner commissure of each eye fissure.**
**exocanthion (ex)**	The point located at the outer commissure of each eye fissure.
**soft tissue nasion (n)**	The mid-point on the soft tissue contour of the base of the nasal root at the level of the frontonasal suture.
**alare (al)**	The most lateral point on each alar contour.
**pronasale (prn)**	The most anterior mid-point of the nasal tip.
**subnasale (sn)**	The mid-point on the nasolabial soft tissue contour between the nasal columella's base and the upper lip.
**labiale superius (ls)**	The mid-point of the vermilion line of the upper lip.
**labiale inferius (li)**	The mid-point of the vermilion line of the upper lip.
**tragion (tr)**	Point located at the upper margin of the left and right tragus.
**glabella (g)**	The most anterior mid-point on the fronto-orbital soft tissue contour.
**soft tissue gonion (go)**	The most lateral point on the soft tissue contour of each mandibular angle located at the intersection of the tangent lines of the posterior border and the inferior border of the margin of the lower face.
**cheilion (ch)**	The point located at each labial commissure.
**sublabiale (sl)**	The most posterior mid-point on the labiomental soft tissue contour defining the border between the lower lip and chin.
**soft tissue pogonion (pog)**	The most anterior mid-point of the bony chin.

Table S2. Linear and angular measurements.

**Total facial height: N-Pg (mm)**	**Vertical linear measurement of facial dimension as measured from nasion (N) to pogonion (Pg)**
**Upper facial height: N-Sn (mm)**	Vertical linear measurement of upper facial dimension as measured from nasion (N) to subnasale (Sn)
**Lower facial height: Sn-Pg (mm)**	Vertical linear measurement of lower facial dimension as measured from subnasale (Sn) to pogonion (Pg)
**Total lip height: Sn-Sl (mm)**	Vertical linear measurement of upper lip dimension as measured for subnasale (Sn) to sublabiale (Sl)
**Vermilion height: Ls-Li (mm)**	Vertical linear measurement of lips from most prominent point of upper lip or labrale superior (Ls) to most prominent point of lower lip or labrale inferior (Li)

**Mouth width: Ch(R)-Ch(L) (mm)**	Transverse linear measurement of mouth width from cheilion right [Ch(R)] to cheilion left [Ch (L)]
**Base nose width: Al(R) – Al(L) (mm)**	Transverse linear measurement of nose width from alar right [Al (R)] to alar left [Al (L)]
**Lateral canthal distance: Ex(R)-Ex(L) (mm)**	Transverse linear measurement of intercanthus width from exocanthion right [Ex (R)] to exocanthion left [Ex (L)]
**Medial canthal distance: En(R)-En(L) (mm)**	Transverse linear measurement of intercanthus width from endocanthion right [En (R)] to endocanthion left [En (L)]
**Nasolabial angle: Prn-Sn-Ls (°)**	Angular measurement of soft-tissue profile measured from pronasale (Prn) to subnasale (Sn) to labiale superior (LS)
**Facial angle: N-Prn-Pg (°)**	Angular measurement of soft-tissue profile measured from nasion (N) to pronasale (Prn) to pogonion (Pg)
**Nasofacial angle: Prn-Sn-Pg (°)**	Angular measurement of soft-tissue profile measured from pronasale (Prn) to subnasale (Sn) to pogonion (Pg)
**Intertragus distance: Tr(L)-Tr(R) (mm)**	Transverse linear measurement measured from tragion right [Tr(R)] to tragion left [Tr (L)]
**Intergonial distance: Go(L)-Go(R) (mm)**	Transverse linear measurement measured from gonion right [Go(R)] to gonion left [Go(L)]
